# Noncanonical-NF-κB activation and DDX3 inhibition reduces the HIV-1 reservoir by elimination of latently infected cells *ex-vivo*

**DOI:** 10.1128/spectrum.03180-23

**Published:** 2023-12-05

**Authors:** Jade Jansen, Stefanie Kroeze, Shirley Man, Matteo Andreini, Jan-Willem Bakker, Claudio Zamperini, Alessia Tarditi, Neeltje A. Kootstra, Teunis B. H. Geijtenbeek

**Affiliations:** 1 Department of Experimental Immunology, Amsterdam UMC Location University of Amsterdam, Amsterdam, the Netherlands; 2 Amsterdam Institute for Infection and Immunity, Amsterdam, the Netherlands; 3 First Health Pharmaceuticals B.V, Amsterdam, the Netherlands; Karolinska Institute, Huddinge, Stockholm, Sweden

**Keywords:** HIV-1 reservoir, human immunodeficiency virus, DDX3, IAP, latency reversal, SMAC mimetics

## Abstract

Latency reversal and subsequent elimination of the human immunodeficiency virus-1 (HIV-1) reservoir using a combination of compounds with different mechanisms of action are considered a promising tool for HIV-1 cure. Here, we analyzed HIV-1 reservoir reduction by targeting the two host factors; inhibitor of apoptosis proteins (IAPs) and DEAD-box polypeptide 3 (DDX3) using a SMAC mimetic (SMACm) and DDX3 inhibitor (DDX3i), respectively. We observed that SMACm efficiently reactivated HIV-1 in a latency Jurkat model, which was further enhanced by DDX3 inhibition. Strikingly, this compound combination strongly decreased the proportion of latently as well as transcriptionally active infected cells in a T cell line model with a dual-reporter virus. To determine the efficacy of compounds to eradicate the HIV-1 reservoir in people living with HIV (PWH), a novel *ex vivo* HIV-1 reservoir reduction assay (HIVRRA) was developed. DDX3i and SMACm alone reduced the HIV-1 reservoir in peripheral blood mononuclear cells (PBMCs) from the majority of PWH, whereas notably, the SMACm/DDX3i combination reduced the HIV-1 reservoir even further with 53%–90% in all PWH analyzed, while uninfected bystander cells were not affected. Our data highlight that IAPs as well as factors involved in HIV-1 replication like DDX3 are excellent targets for HIV-1 cure strategies. We show for the first time that the combination of SMACm and DDX3i reverses viral latency and specifically eliminates the HIV-1-infected cells *in vitro* and *ex vivo*.

**IMPORTANCE:**

HIV-1 continues to be a major global health challenge. Current HIV-1 treatments are effective but need lifelong adherence. An HIV-1 cure should eliminate the latent viral reservoir that persists in people living with HIV-1. Different methods have been investigated that focus on reactivation and subsequent elimination of the HIV-1 reservoir, and it is becoming clear that a combination of compounds with different mechanisms of actions might be more effective. Here, we target two host factors, inhibitor of apoptosis proteins that control apoptosis and the DEAD-box helicase DDX3, facilitating HIV mRNA transport/translation. We show that targeting of these host factors with SMAC mimetics and DDX3 inhibitors induce reversal of viral latency and eliminate HIV-1-infected cells *in vitro* and *ex vivo*.

## INTRODUCTION

Persistence of the human immunodeficiency virus-1 (HIV-1) reservoir in people living with HIV (PWH) on combination antiretroviral therapy (cART) remains the biggest obstacle to a cure. The HIV-1 reservoir does not decline over the years despite long-term effective cART, suggesting a highly dynamic HIV-1 reservoir that is partially maintained by ongoing low-level residual viral replication (1 – 4). Moreover, integrated proviruses can persist in long-lived CD4 +T cells, establishing a latent reservoir that can facilitate viral rebound upon cART cessation (2, 5, 6). These long-lived infected cells are maintained through clonal expansion, thus sustaining the viral reservoir (7). Diverse viral integration sites, infection of various cell types, and transcriptional modifications further contribute to the immense heterogeneity of the latent reservoir, making elimination of the viral reservoir very complex (8).

Several cure strategies are being explored, such as the “shock-and-kill” strategy that aims to activate proviral transcription with a latency reversing agent (LRA) and subsequently kill the reactivated cells via immune-mediated pathways or virus-induced cytolysis (9, 10). Thus far, “shock-and-kill” research has primarly focussed on the development and discovery of novel LRAs. Many LRAs target host-dependent mechanisms that play a role in viral latency or transcription and have proven to be successful *in vitro*, such as histone deacetylase inhibitors, protein kinase C (PKC) agonists, and toll-like receptor agonists (11 – 13). However, latency reversal does not always lead to effective clearance of the viral reservoir *in vivo* (14, 15) due to the inability to induce cell death after reactivation and the heterogenous nature of the latent reservoir. Therefore, a combination of compounds targeting various host proteins to induce reactivation and also to eliminate the reactivated reservoir might be more effective.

HIV-1 transcription is regulated by host cell transcripton factors of which nuclear factor kappa-light-chain-enhancer of activated B cells (NF-κB) is a crucial initiator (9, 16). PKC agonists such as phorbol esters and ingenol derivatives that activate the canonical (c)NF-κB pathway have proven to be potent LRAs. However, the broad activity of these compounds may also trigger toxicity due to systemic inflammation and hypercytokinemia (12, 17, 18). Alternatively, activation of the noncanonical (nc)NF-κB pathway may limit the risk of systemic inflammation, as this pathway is slow and activates fewer genes compared to the canonical pathway. Inhibitor of apoptosis proteins (IAPs) are negative regulators of the ncNF-κB pathway. Activation of the ncNF-κB pathway is dependent on NF-κB-inducing kinase (NIK), a central regulator within this pathway. NIK is continuously targeted for ubiquitination by the inhibitory IAPs complex, thus preventing NIK accumulation and ncNF-κB activation (19, 20). The second mitochondrial-derived activator of caspase mimetics (SMACm) prevents ubiquitination of NIK through targeting of the IAP inhibitory complex, thereby leading to activation of ncNF-κB (20, 21). Previous research showed that activation of the ncNF-κB pathway with SMACm enhanced HIV-1 transcription leading to latency reversal in the J-lat HIV-1 latency model (22) as well as *in vivo* (23). DEAD-box polypeptide 3 (DDX3) has also been suggested as a potential target for latency reversal (24). Host factor DDX3 is an RNA helicase involved in Rev-dependent nucleocytoplasmic shuttling of HIV-1 mRNA and translation of viral proteins (25 – 30).

In this study, we investigated the potency of SMACm AZD5582 in combination with DDX3i FH1321 to reverse viral latency and to induce death of the infected cell. Our data strongly suggest that the combination of SMACm and DDX3i leads to reactivation of latent HIV-1 as well as reduction of the HIV-1 reservoir as shown by *in vitro* assays and our innovative *ex vivo* HIV-1 reservoir reduction assay using peripheral blood mononuclear cells (PBMCs) from PWH on cART.

## RESULTS

### DDX3 inhibition enhances SMACm-induced reactivation of the latent HIV-1 provirus

**Fig 1:**
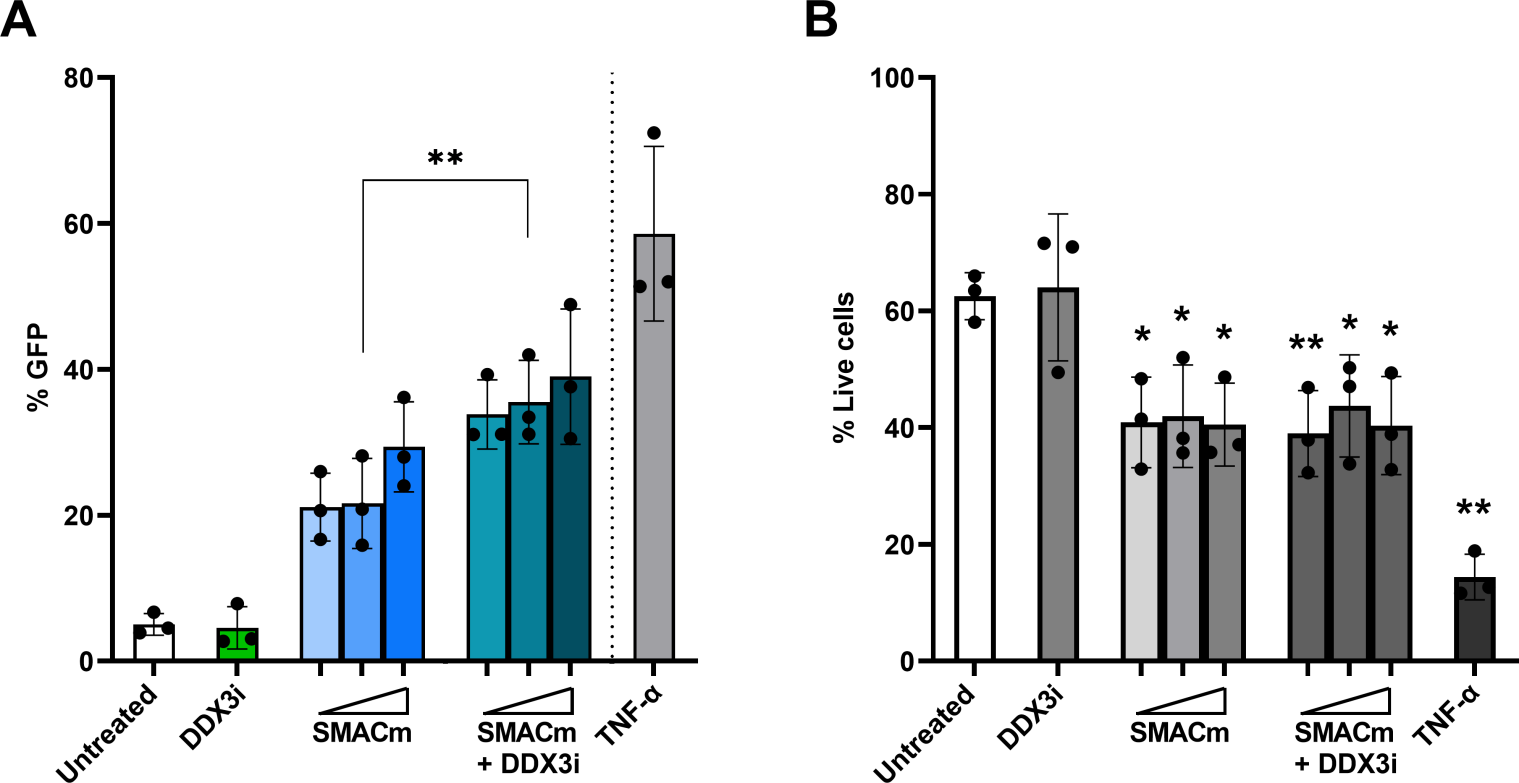
DDX3 inhibition enhances SMACm-induced reactivation of the latent HIV-provirus. J-Lat A1 cells were treated with SMACm AZD5582 (0.2–5 µM), DDX3i FH1321 (75 µM), or both for 2 days, and reversal of viral latency was analyzed by GFP expression (**A**). Cell toxicity was analyzed by live/dead staining (**B**). Each graph displays the mean and standard deviation of three independent experiments. Comparisons for SMACm only to SMACm in combination with DDX3i (**A**) or each concentration to untreated (**B**) were made using a paired *t* test. Only significant events are displayed, *P < 0.05, **P < 0.01.

Here, we investigated whether SMACm and DDX3i are able to reactivate the HIV-1 provirus in the J-lat A1 and J-lat 10.6 latency cell models, which contain green fluorescent protein (GFP) under control of the long terminal repeat (LTR) allowing quantification of LTR-driven transcription (31, 32). Increasing concentrations of SMACm resulted in a dose-dependent increase of GFP-expressing cells after 48 hours of treatment onset (Fig. 1A; Fig. S1), indicating that SMACm induces viral reactivation. DDX3i alone had no effect on GFP expression (Fig. 1A). Notably, combining DDX3i with SMACm resulted in a significant increase in HIV-1 reactivation compared to SMACm or DDX3i alone (Fig. S1). In contrast to DDX3i, SMACm alone as well as in combination with DDX3i significantly reduced cell viability in the J-lat A1 cells compared to untreated cells (Fig. 1B). These data suggest that SMACm reactivates HIV-1 transcription in the J-lat A1 cell model, and viral reactivation is further enhanced by DDX3i.

### Synergistic effect of DDX3i and SMACm on cell death of HIV-1-infected cells

**Fig 2:**
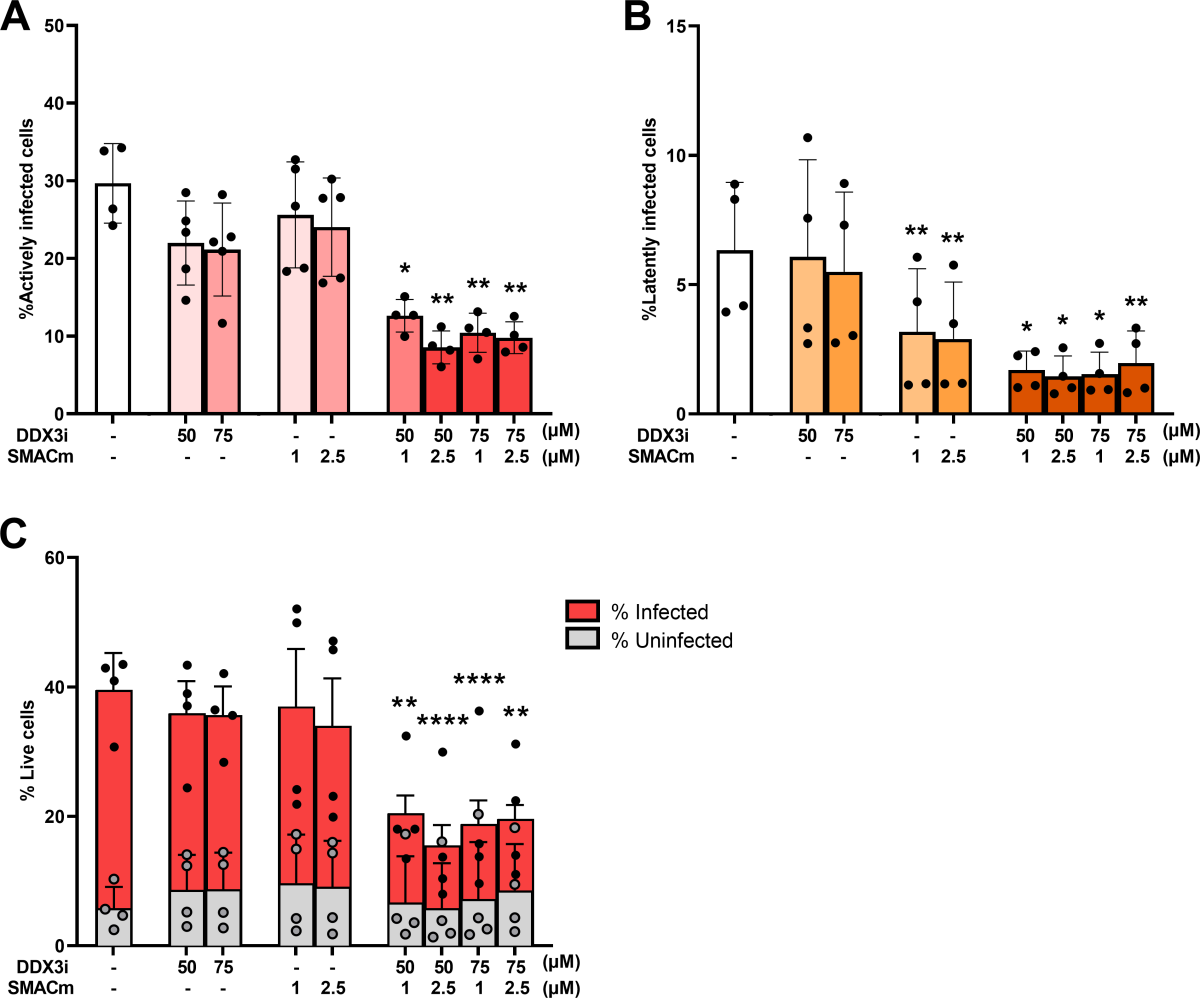
Synergistic effect of DDX3i and SMACm on cell death of HIV-1-infected cells. SUPT1-CCR5 cells were infected with a dual-reporter virus HIV-GKO-VSV-G and subsequently treated with SMACm AZD5582 (1 and 2.5 µM), DDX3i FH1321 (50 and 75 µM), or both for 2 days. The proportion of infected cells containing a transcriptionally active provirus (**A**) or a latent provirus (**B**) was analyzed by flow cytometry, as well as cell viability staining with a live/dead marker (**C**). Each graph displays the mean and standard deviation of three independent experiments. Comparisons for each concentration to untreated were made using a paired *t* test. Only significant events are displayed, *P < 0.05, **P < 0.01, ***P < 0.001, ****P < 0.0001.

Next, we investigated the effect of DDX3i and SMACm on transcriptionally latent and active HIV-1-infected cells using the dual-reporter virus HIV-GKO pseudotyped with a vesicular stomatitis virus glycoprotein (VSV-G) allowing identification of infection and viral transcription by analyzing the expression of EF1α-driven mKusabira-Orange2 (mKO2) and HIV-1 LTR-driven GFP, respectively. SUPT1-CCR5 cells were infected with the dual-reporter virus, and compounds were added 24 hours after infection. Two days after addition of the compounds, infection status was analyzed by flow cytometry (Fig. S2). The proportion of mKO2/GFP-double positive cells, representing cells with transcriptionally active provirus, slightly decreased in the presence of either DDX3i or SMACm (Fig. 2A). However, a strong significant decrease of double-positive cells was observed with the combination of DDX3i and SMACm. The proportion of transcriptional latently infected mKO2-positive cells significantly decreased by SMACm treatment as well as the combination of DDX3i and SMACm but not by DDX3i alone (Fig. 2B). Next, we analyzed the effect of the compounds on the viability of the infected and uninfected cell population. Notably, in line with the effect on the proportion of active and latent cells (Fig. 2A and B), we observed a strong and significant decrease in viability of HIV-1-infected cells treated with the combination of SMACm and DDX3i, in contrast to each compound alone (Fig. 2C). Viability of uninfected cells was neither affected by the single compounds nor the combination (Fig. 2C; Fig. S3). Induction of apoptosis by the compounds was measured in SUPT1-CCR5 cells infected with HIV-1_BAL_ for 2 days, and we observed increased cell death in the HIV-1-infected (p24+) population in the presence of the compounds, whereas the uninfected population (p24-) was not affected (Fig. S4). Our data strongly suggest that the combination of SMACm and DDX3i specifically induces cell death of HIV-1-infected cells.

### Inducible HIV-1 reservoir reduction assay

**Fig 3:**
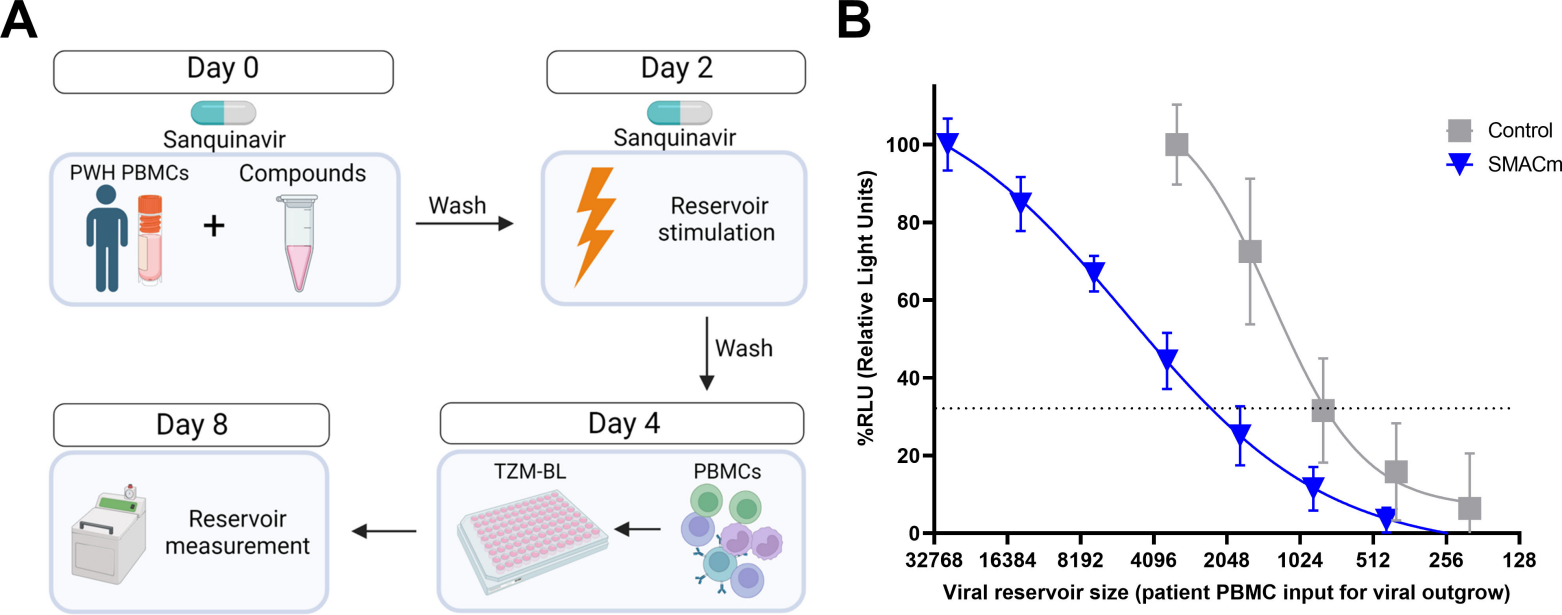
HIVRRA to measure the inducible replication competent HIV-1 reservoir in PBMCs *ex vivo*. Schematic representation of the HIVRRA (**A**). Representative logistic regression plot of the quantification of HIV-1-infected cells in PBMC from PWH using TZM-BL for SMACm AZD5582 (0.02 µM) and DMSO control (**B**).

To determine the effectivity of the compounds to reduce the size of the viral reservoir *ex vivo*, using PBMCs from PWH, we designed a new inducible HIV-1 reservoir reduction assay (HIVRRA) (Fig. 3A). In brief, PBMCs from PWH on cART were cultured in the presence of SMACm or control (dimethyl sulfoxide [DMSO]) for 2 days. To prevent spreading of virus produced upon reactivation, the protease inhibitor Saquinavir was added. To establish cell survival after compound addition, the cultures were spiked with a fixed amount of CellTrace Violet (CTV)-labeled healthy donor PBMCs. The ratio of labeled (PBMC donors) and unlabeled (PBMC PWH) cells was determined by flow cytometry, and similar proportions of 52.4% and 52.3% of unlabeled PBMC from PWH were observed after treatment with SMACm or DMSO, respectively (Fig. S5). Subsequently, cells were stimulated with phytohemagglutinin (PHA) in the presence of Saquinavir for 2 days to induce virus production, and the number of HIV-1-infected cells in the culture was determined by a modified TZM-BL-based assay (TZA) (33). The relative infectious units per million (IUPM) cells were calculated by logistic regression (Fig. 3B). Using the HIVRRA, a 2.8-fold reduction of the HIV-1 reservoir upon SMACm treatment was observed.

### The combination of SMACm and DDX3i reduces the inducible HIV-1 reservoir in PBMC from PWH *ex vivo*

Using the HIVRRA, we investigated the effect of the SMACm and DDX3i alone or in combination on the inducible HIV-1 reservoir in PBMCs from PWH on effective cART *ex vivo*. We selected 10 participants from the Amsterdam Cohort Studies (ACS) (Table 1); however, eventually, nine participants were included in the analysis as the assay failed for one participant. Participants were all male and on effective cART for at least 8 months. All participants had an undetectable viral load for at least 6 months and their CD4 +T cell count recovered to >400 cells/mm^3^.

**TABLE 1:** Participant characteristics

Characteristic	Value
Total participants	10
Gender: male	100%
Age at time of sampling (years, range)	44 (38–54)
CD4 +T cell count nadir (cells/mm^3^, range)	163 (30–280)
Plasma viral load before cART initiation in copies/mL, range)	69,870 (22,222–162,814)
Time on effective cART (months, range)	21,5 (8 – 30)
Time of undetectable viral load (months, range)^a^	19,21 (6 – 27)
CD4 +T cell count upon sampling (cells/mm^3^, range)	580 (420–830)
Start cART (year, range)	1996 (1996–1997)
cART regimen	
Combination of (N)NRTI and PI	9
Combination of (N)NRTI	1

^a^ Months between the time point of first undetectable viral load and the sample analyzed. NRTI: Nucleoside reverse transcriptase inhibitor, NNRTI: Non-nucleoside reverse transcriptase inhibitor, PI: Protease inhibitor.

**Fig 4:**
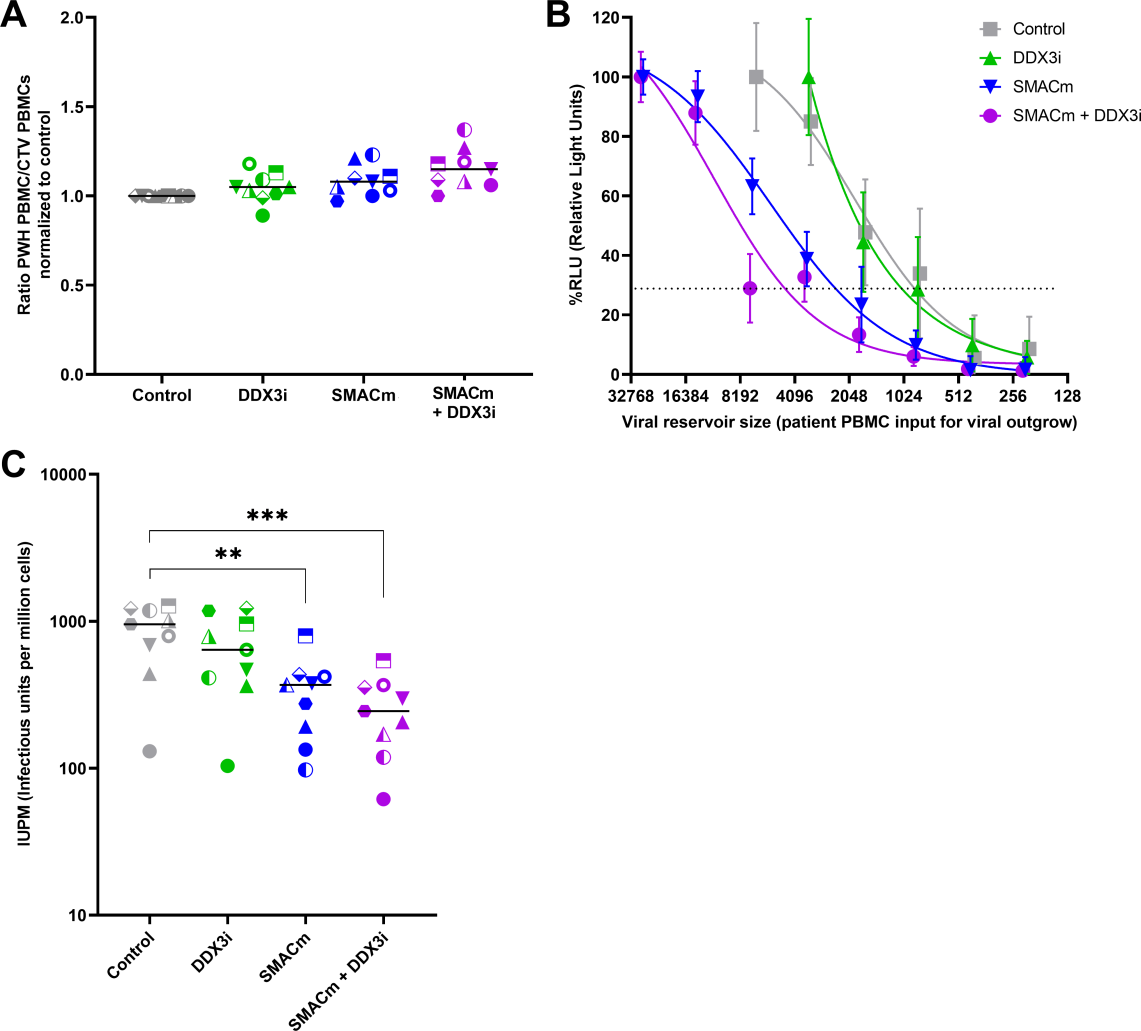
The combination of SMACm and DDX3i reduces the inducible HIV-1 reservoir in PBMC from PWH *ex vivo.* Ratio of PWH PBMCs/healthy CTV-stained PBMCs normalized to DMSO samples is displayed to check for cell survival of the compounds; the median is displayed (**A**). Logistic regression plot of the quantification of HIV-1-infected cells in PBMC from PWH after treatment with SMACm AZD5582, DDX3i FH1321, both, or DMSO control using TZM-BL cells (**B**). IUPM after treatment with the compounds for each participant; the median IUPM is displayed. Comparisons of each condition to control were made using a paired *t* test (**C**). Significant differences are indicated, **P < 0.01, ***P < 0.001.

The proportion of PBMCs from PWH did neither decrease after treatment with DDX3i and SMACm alone nor in combination compared to the control (Fig. 4A), indicating that the compounds were not toxic at the concentrations used. Using logistic regression analysis, the IUPM was calculated for each condition for every participant (Fig. 4B). PWH PBMCs treated with only DMSO displayed the highest IUPM overall (range: 131–1272, median IUPM: 954). DDX3i treatment of PBMCs from PWH resulted in a small reduction in the HIV-1 reservoir in seven out of nine PWH, and the reservoir size ranging from 103 to 1229 IUPM with a median IUPM of 640.3 was found. Treatment with the SMACm resulted in a reduction in the reservoir in eight out of nine PWH. Overall, the reservoir size ranged from 97 to 795 IUPM (median: 368.5) after treatment with SMACm, indicating a 0%–91% reduction in the viral reservoir. Combination of SMACm and DDX3i decreased the reservoir in all PWH (range 61–538 IUPM, median IUPM: 244.8). Interestingly, SMACm in combination with DDX3i significantly reduced the viral reservoir ranging from 53% to 90% (Fig. 4C) in *ex vivo* PBMC from PWH on cART. These data indicate that the combination of SMACm and DDX3i induces cell death specifically in the infected cells from PWH.

## DISCUSSION

Investigating different strategies to reduce the viral reservoir in PWH is crucial for HIV-1 cure research. Single LRAs so far have proven efficacy *in vitro* but not *in vivo* in PWH with regard to latency reversal as well as reduction in the viral reservoir, and therefore, a combination of multiple compounds is likely to be more effective (34). Here, we show that SMACm and DDX3i act synergistically and the combination enhances latency reversal and induces specific elimination of transcriptionally active as well as latently infected cells *in vitr*o, as compared to each compound alone. Notably, the combination of SMACm and DDX3i effectively reduced the inducible HIV-1 reservoir in PBMC from PWH *ex vivo*. These data strongly suggest that targeting the ncNF-κB pathway with SMACm and transcription/transport machinery with DDX3i might be an effective strategy to reactivate and eliminate the HIV-1 reservoir in PWH.

Both the SMACm and DDX3i used in this study have been shown to induce HIV-1 reactivation (23, 24, 35). Our data support the notion that SMACm can act as an LRA in J-lat A1 cell line, which mimics HIV-1 viral latency; however, contradicting results were obtained using DDX3i that may be related to the compound-protein interaction site. In our study, the DDX3i FH1321, which targets the DDX3 RNA-binding site, was unable to reverse viral latency unaided in the J-lat clones used, while Rao et al. showed latency reversal in similar models using the DDX3i RK-33, which blocks the ATP-binding site. However, differential LRA sensitivity between J-Lat clones has previously been observed (36), which may explain differences between the studies. Notably, we observed a synergistic increase of latency reversal when DDX3 inhibition was combined with the SMACm in the J-lat cells. SMACm leads to activation of the ncNF-κB pathway, and our data suggest that this induces HIV-1 transcription. DDX3 has been shown to suppress the canonical NF-κB pathway (37), and therefore, DDX3 inhibition might enhance the SMACm-induced HIV-1 reactivation through activation of the cNF-κB pathway.

Our results suggest that SMACm AZD5582 not only acts as an LRA but also induces apoptosis of infected cells, in particular in combination with DDX3i. AZD5582 was first developed as cancer treatment to restore sensitivity to apoptotic stimuli as some cancer types would overexpress IAP proteins (38). AZD5582 targets these IAPs by binding to the BIR3 domains of cIAP1, cIAP2, and XIAP, inhibiting their anti-apoptotic function. cIAP1 and cIAP2 can inhibit apoptosis through ubiquitination of NF-κB-inducing kinase (NIK) involved in the ncNF-κB pathway and activation of caspase-8 through TNF receptor signaling (38, 39). XIAP can bind pro-caspases directly and prevent them from becoming active (40, 41). Studies have shown upregulation of XIAP, cIAP1, and cIAP2 in HIV-1-infected macrophages and latently infected cell lines, suggesting an important role of IAPs in the survival of HIV-1-infected cells (42 – 46). Thus, AZD5582 promotes latency reversal and blocks anti-apoptotic IAP proteins, resulting in a selective death of HIV-1 infected cells.

Combining DDX3i together with SMACm resulted in increased apoptosis of HIV-1-infected cells, whereas the uninfected cells remained unaffected. This suggests a role for DDX3i in the induction of apoptosis, specifically in HIV-1-infected cells. Previous research showed that DDX3 is a key player in the nucleocytoplasmic shuttling complex required in the Rev-mediated transport of partially spliced and unspliced HIV-1 RNAs from the nucleus to the cytoplasm (25 – 30). Treatment with DDX3i could therefore lead to accumulation of HIV-1 RNAs inducing apoptosis, especially in SMACm-treated cells as these are sensitized to apoptosis by inhibition of anti-apoptotic IAPS. Interestingly, the combination did not induce cell death in uninfected cells as well as in PBMCs from PWH, indicating that the combination treatment is specific for infected cells. Further studies are required to identify the mechanism of the synergistic action between SMACm and DDX3i with regard to both latency reversal and specific elimination of HIV-1-infected cells. Notably, the increased activity of SMACm and DDX3i observed in primary PBMCs from PWH as compared to *in vitro* latency models suggests that the *in vivo* HIV-1 reservoir will be sensitive to these compounds already at low concentrations. Our data show that DDX3 inhibition enhances SMACm-induced HIV-1 reservoir reduction in *in vitro* reservoir models and in *ex vivo* PBMC from PWH, thus indicating that targeting of host factors involved in viral replication, cellular apoptotic processes as well as transcriptional regulation holds promise for future HIV-1 cure strategies.

## MATERIALS AND METHODS

### Cell cultures

J-lat cells (clone A1 and 10.6) and SUPT1-CCR5 cells were obtained through the NIH HIV Reagent Program, Division of AIDS, NIAID, NIH. The J-Lat Tat-GFP clone A1 is a Jurkat cell harboring an integrated HIV-1 LTR driving Tat and GFP expression. J-lat full-length clone 10.6 is a Jurkat cell line harboring a full-length integrated HIV genome with a frameshift in the envelope gene and a GFP reporter in the nef openreading frame (31, 32). The T-cell line SUPT1 was lentivirally transduced to express CCR5 (47). Cell lines were cultured in Iscove’s Modified Dulbecco’s Medium (IMDM; Thermo Fisher Scientific, Gibco) supplemented with 10% fetal calf serum (FCS; HyClone, Cytiva, Marlborough, MA, USA) and antibiotics (100 U/mL penicillin and 100 ug/mL streptomycin) (Invitrogen, Carlsbad, CA, USA) at 37°C in a humidified 10% CO_2_ incubator. HEK293T (ATCC Cat# CRL-3216) were cultured and maintained in Dulbecco’s Modified Eagle Medium (DMEM; Thermo Fisher Scientific, Gibco) supplemented with 10% inactivated FCS and antibiotics, in a humidified 10% CO_2_ incubator at 37°C. Peripheral blood mononuclear cells (PBMCs) were obtained from blood bank donors (Sanquin, Amsterdam, the Netherlands) and cultured in IMDM supplemented with 10% FCS, antibiotics (100 U/mL penicillin, 100 ug/mL streptomycin and ciproxine 5 µg/mL) and 20 U/mL IL-2.

### Compounds

AZD5582 (Tebu-Bio, Le Perray en Yvelines, France) was dissolved in DMSO at 10 mM concentration and stored at −80°C. Before use, AZD5582 was diluted in a culture medium to a final concentration of 0.02 µM-5µM. DDX3i FH1321 was provided by First Health Pharmaceuticals (Amsterdam, the Netherlands) and dissolved in DMSO at concentrations of 20 mM. Before use, DDX3i was diluted in a culture medium to a final concentration ranging from 10 to 75 µM. TNF-α (PeproTech, Londen, United Kingdom) was dissolved in a medium at 500 ng/mL concentration and stored at −20°C until further use. Upon use, TNF-α was diluted to 50 ng/mL in the culture medium.

### Virus production and infection assays

The dual-reporter virus, HIV-GKO (48) pseudotyped with a vesicular stomatitis virus glycoprotein (HIV-GKO_VSV-G_), and HIV-1_BAL_ were produced by (co-)transfection of HEK293T cells using the calcium phosphate method. Briefly, for a six-well plate, plasmid DNA was diluted in 0.042M HEPES containing 0.15M CaCl_2_ and subsequently carefully mixed with an equal volume of 2 x HBS (HEPES-buffered saline). After 15 minutes, the mixture was added dropwise to HEK293T cells followed by overnight incubation at 37°C in a humidified 3% CO_2_ incubator. The following day, the culture medium was replaced, and HEK293T cell cultures were continued at 10% CO_2_ at 37°C. Virus was harvested at days 2 and 3 after transfection, passed through a 0.22 µm filter and stored in aliquots at −80°C for later use. Virus titers were quantified with TZM-BL cells, determining the TCID50. Before use, virus stocks were treated with DNase (Promega, Madison, WI, USA) for 30 minutes at 37°C to eliminate any residual plasmid DNA.

SUPT1-CCR5 cells were infected with HIV-GKO_VSV-G_ (MOI 0.3), and after 2 days, viral infection and transcriptional activity were determined by flow cytometry: EF1α-driven mKO2 expression determines HIV-1 infection and HIV-1 LTR-driven GFP expression is indicative of the viral transcription activity.

SUPT1-CCR5 cells were infected with HIV-1_BAL_ (MOI0.5), and after 2 days, viral infection and induction of apoptosis were determined by flow cytometry.

### Study population

Ten participants from the Amsterdam Cohort Studies (ACS) on HIV-1 infection and AIDS were selected for this study. These participants entered the cohort after HIV-1 seroconversion and had an untreated course of infection of at least 2 years before the initiation of suppressive cART.

### Inducible HIV-1 reservoir reduction assay (HIVRRA)

The frequency of inducible HIV-1-infected CD4 +T cells from PWH was determined by the HIVRRA assay. In short, PBMCs from PWH were treated with 10 µM DDX3i and 0.02 µM AZD5582 alone or in combination for 2 days in the presence of 10 µM Saquinavir. Subsequently, PBMCs were stimulated with 1 µg/mL PHA in the presence of Saquinavir. After 2 days, PBMCs were washed and seeded in an 11-fold titration containing 3 × 10^4^ PBMCs in the first row and serially diluted 1:2 across a 96-well plate onto 3 × 10^4^ TZM-BL cells. Virus production was measured with the TZA.

To determine cytotoxicity of the compounds during the assay, a set amount of CTV-labeled PBMCs from blood donors were used as spike-in, and the ratio between CTV-labeled PBMC and unlabeled PWH PBMC was determined by flow cytometry. This also allowed to correct for PWH PBMC cell input for downstream reservoir calculations.

### TZM-BL based assay (TZA)

The TZA assay (33) was slightly modified to measure virus production from PWH PBMCs. PBMCs from the HIVRRA assay were co-cultured with TZM-BL cells in a 96-well plate for 4 days. LTR-driven luciferase activity from TZM-BL cells co-cultured with PBMCs from PWH was measured by addition of 25 µL of luciferase activity reagent (LAR) substrate (0.83 mM ATP, 0.83 mM of d-Luciferin [Duchefa Biochemie B.V., Haarlem, the Netherlands], 18.7 mM MgCl_2_, 0.78 µM Na2H2P2O7, 38.9 mM Tris [pH 7.8], 0.39% glycerol, 0.03% Triton X-100, and 2.6 µM dithiothreitol) and measuring luminescence in relative light units (RLUs) using a luminometer (Berthold Technologies, Germany). The relative infectious units per million (IUPM) cells were calculated at 30% of the maximum RLU using logistic regression.

### Flow cytometry

J-lat A1 and SUPT1-CCR5 (with or without HIV-GKO_VSV-G_ infection) were washed with phosphate-buffered saline (PBS, Thermo Fisher Scientific, Gibco) and stained with a fixable live/dead marker (Invitrogen, Carlsbad, CA, USA) for 30 minutes at 4°C in the dark. Cells were washed twice with PBS and fixed using BD CellFIX (BD Biosciences, Franklin Lakes, NJ, USA). PBMCs were stained with CellTrace Violet (Thermo Fisher Scientific, Gibco) according to manufacturer’s protocol and fixed afterward. SUPT1-CCR5 cells infected with HIV-1_BAL_ were stained with FLICA 660 Caspase-3/7 Kit (Biorad, Hercules, CA, USA) and a fixable live/dead marker according to manufacturer’s protocol, fixed, and thereafter permeabilized with BD Cytofix/Cytoperm solution (BD Biosciences, Franklin Lakes, NJ, USA) according to manufacturer’s protocol and stained intracellular for p24 (HIV-1 core antigen-FITC KC57) (Beckman Coulter, Indianapolis, IN, USA). Fluorescence was measured with the BD LSRFortessa (BD Biosciences, Franklin Lakes, NJ, USA). Flow cytometry data were analyzed using FlowJo version 10 (Treestar, Ashland, OR, USA).

### Statistics

Data were analyzed using Graphpad Prism 9.3.1 (Graphpad software Inc., San Diego, CA, USA). Differences between two groups were determined using a paired student *t* test when data were normally distributed, or the Wilcoxon matched-pairs signed-rank test. Logistic regression was used to determine the frequency of HIV-1-infected cells in the HIVRRA. A p value of < 0.05 was considered statistically significant.

## Data Availability

Supporting data are available in the supplemental material.

## SUPPLEMENTAL MATERIAL

Supplemental figures spectrum.03180-23-s0001.docx Figures S1 to S5.

ASM does not own the copyrights to Supplemental Material that may be linked to, or accessed through, an article. The authors have granted ASM a non-exclusive, world-wide license to publish the Supplemental Material files. Please contact the corresponding author directly for reuse.
