# Supplementary material for: Noncanonical-NF-κB activation and DDX3 inhibition reduces the HIV-1 reservoir by elimination of latently infected cells ex-vivo
Source: Microbiol Spectr. 2023 Dec 5;12(1):e03180-23. doi: 10.1128/spectrum.03180-23 (PMC10783037; doi:10.1128/spectrum.03180-23)
Supplement: Supplemental figures — Figures S1 to S5. [file spectrum.03180-23-s0001.docx]

**Figure S1**. J-lat 10.6 cells were treated with DMSO, DDX3i (10, 50 or 75uM), SMACm (0.2, 1 or 5uM) or both for two days and reversal of viral latency was analysed by GFP expression. Representative figure of 4 experiments.


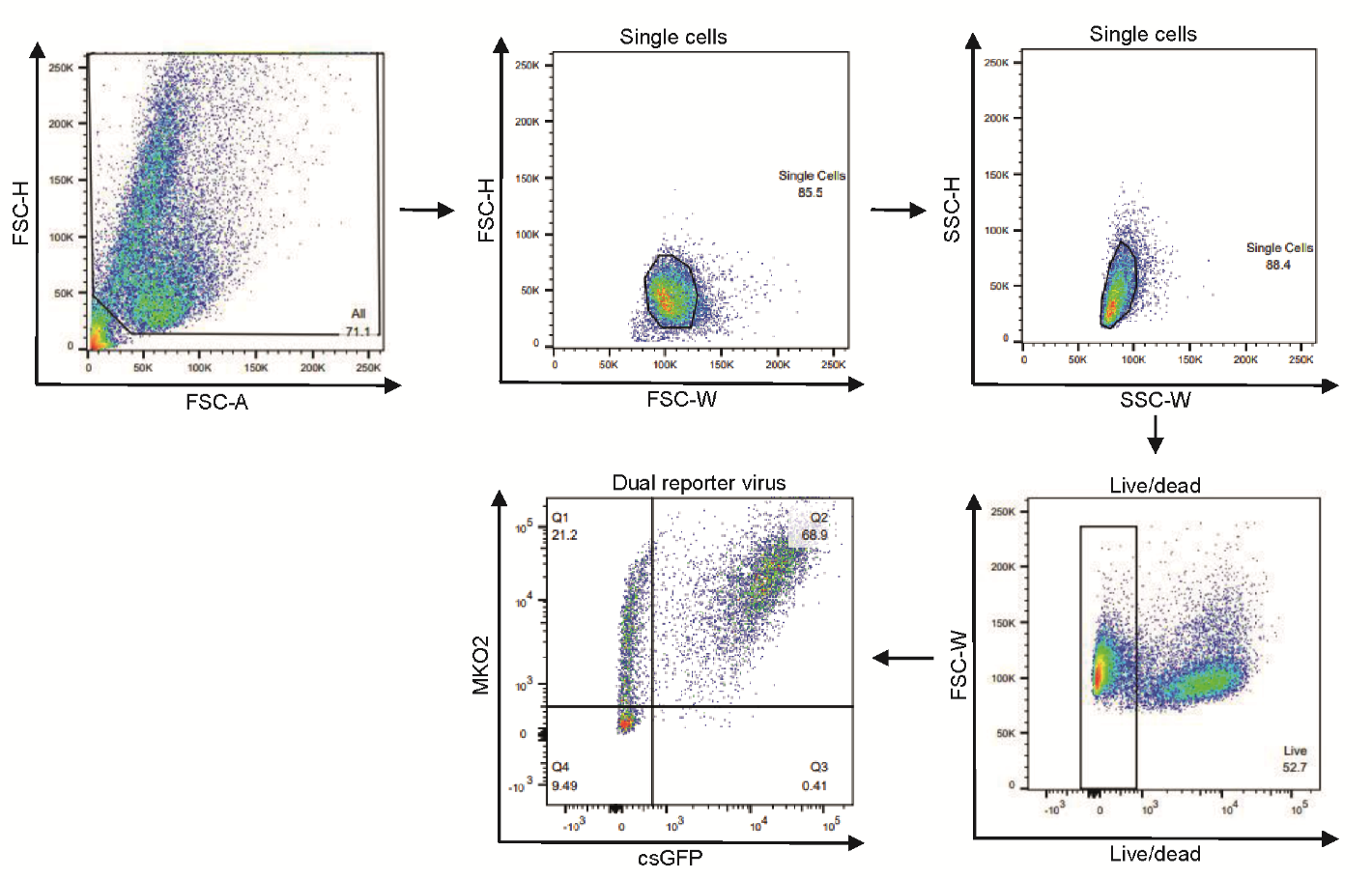


**Figure S2.** Representative gating strategy of SUPT1-CCR5 infected with HIV-GKO-VSV-G dual reporter virus, distinguishing a transcriptionally active provirus (expressing both EF1α-driven mKO2 and LTR-driven GFP) from a transcriptionally latent provirus (expressing only EF1α-driven mKO2) untreated.


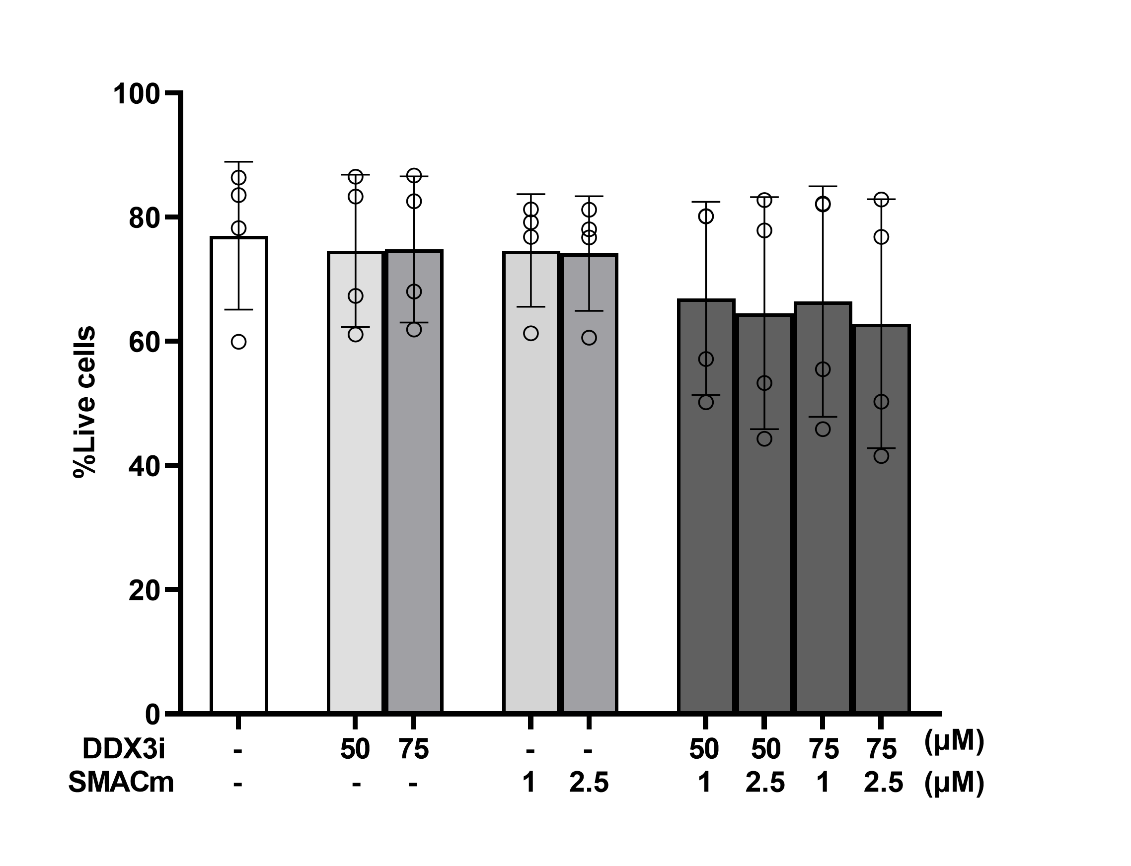


**Figure S3.** SUPT1-CCR5 cells were treated with DDX3i FH1321 (50-75µM), SMACm AZD5582 (1 and 2.5µM) or a combination of both and cell toxicity was analyzed by live/dead staining.


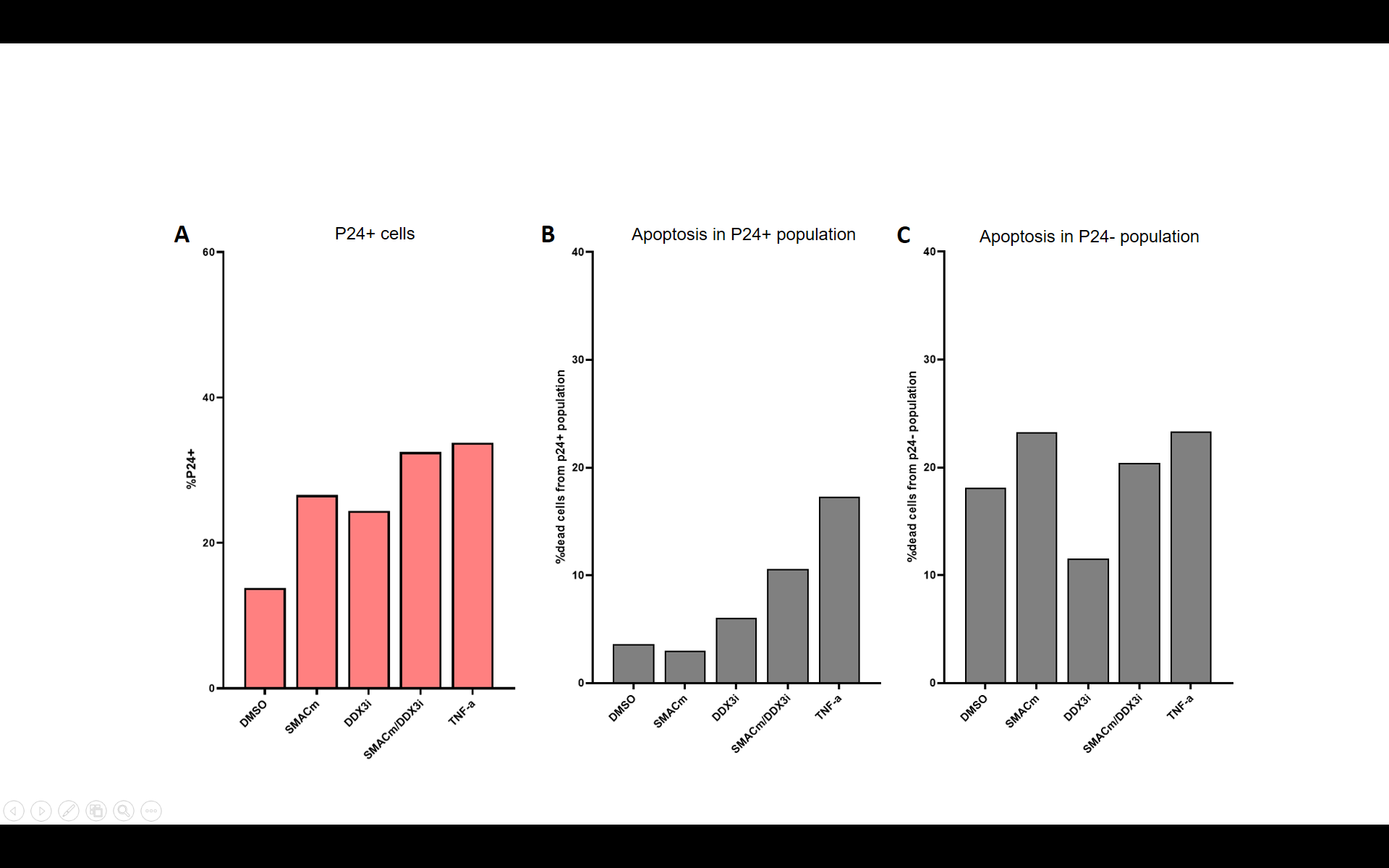


**Figure S4**. SUPT1 cells infected with HIV-1_BAL_ were treated with DMSO, TNF-α, SMACm, DDX3i. (A) Percentage of p24 positive cells as determined by flow cytometry; (B) Percentage of dead-cells in the uninfected population (p24-negative) as measured by Caspase-3/7 and live/dead staining; (C) Percentage of dead-cells in the infected population (p24-positive) as measured by Caspase-3/7 and live/dead staining.


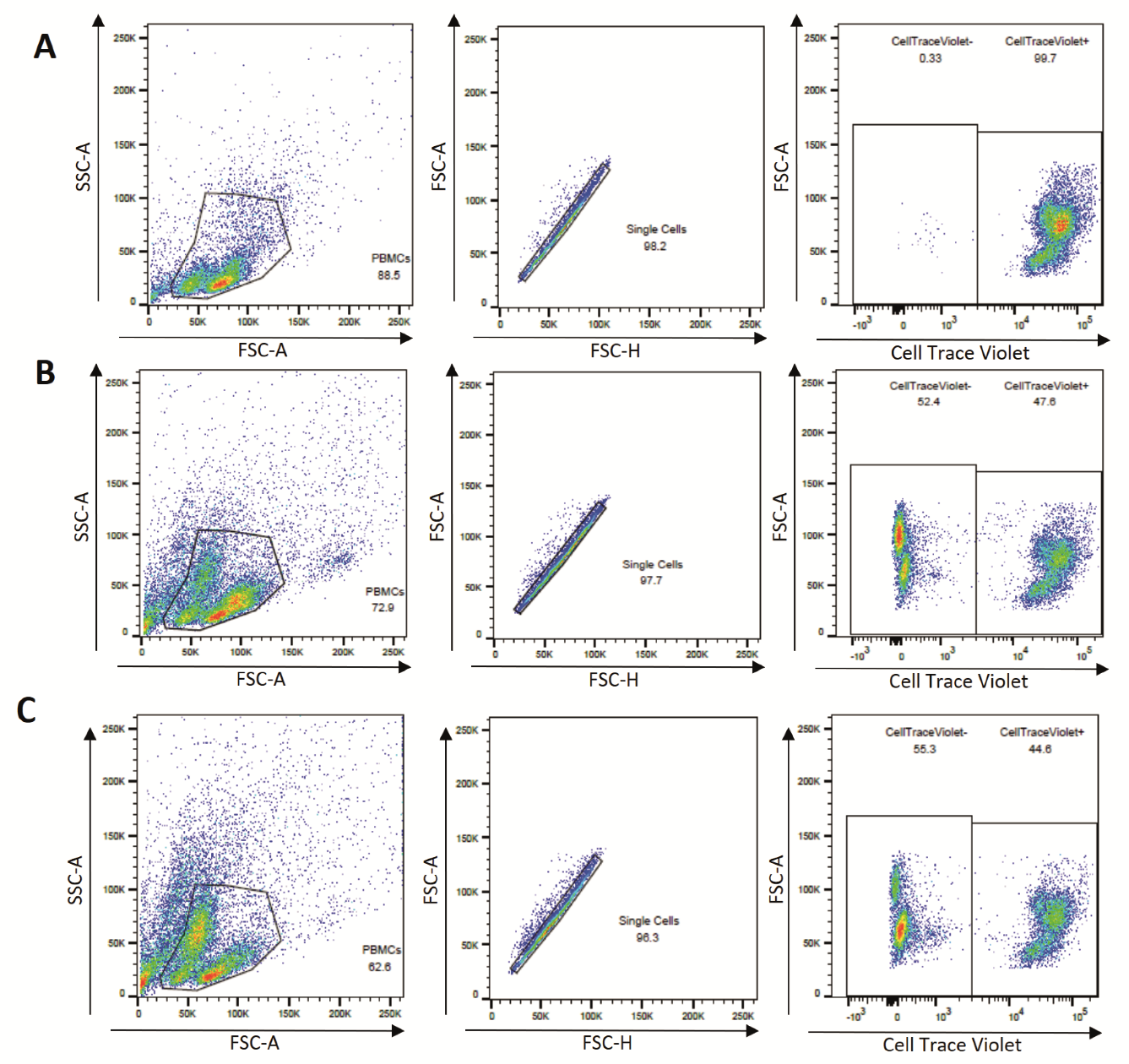
 **Figure S5.** Assessment of cytotoxicity of the compounds with CTV stained healthy PBMCs. PBMCs from PWH were spiked 1:1 with healthy CTV stained PBMCs after two day treatment with the compounds or vehicle control DMSO. A cell sample was taken and analysed by flow cytometry. Flow cytometry gating strategy of healthy PBMCs stained with CTV (A) and representative gating strategy of PBMCs from PWH treated with DMSO spiked in with CTV PBMCs (B) and for SMACm (C).
